# Functional mutants of *Azospirillum brasilense* elicit beneficial physiological and metabolic responses in *Zea mays* contributing to increased host iron assimilation

**DOI:** 10.1038/s41396-020-00866-x

**Published:** 2021-01-06

**Authors:** A. B. Housh, G. Powell, S. Scott, A. Anstaett, A. Gerheart, M. Benoit, S. Waller, A. Powell, J. M. Guthrie, B. Higgins, S. L. Wilder, M. J. Schueller, R. A. Ferrieri

**Affiliations:** 1 Missouri Research Reactor Center, University of Missouri, Columbia, MO 65211 USA; 2 Chemistry Department, University of Missouri, Columbia, MO 65211 USA; 3 Department of Biochemistry, University of Missouri, Columbia, MO 65211 USA; 4 Department of Chemical Engineering, University of Missouri, Columbia, MO 65211 USA; 5 Division of Plant Sciences, University of Missouri, Columbia, MO 65211 USA; 6 School of Natural Resources, University of Missouri, Columbia, MO 65211 USA; 7 Interdisciplinary Plant Group, University of Missouri, Columbia, MO 65211 USA; 8 Present Address: Burns & McDonnell, Inc. 425 S, Woods Mill Rd., Chesterfield, MO USA 63017; 9 Present Address: Idaho State Police 5255 S. 5th Ave, Pocatello, ID 83204 USA

**Keywords:** Plant sciences, Applied microbiology

## Abstract

Iron (Fe), an essential element for plant growth, is abundant in soil but with low bioavailability. Thus, plants developed specialized mechanisms to sequester the element. Beneficial microbes have recently become a favored method to promote plant growth through increased uptake of essential micronutrients, like Fe, yet little is known of their mechanisms of action. Functional mutants of the epiphytic bacterium *Azospirillum brasilense*, a prolific grass-root colonizer, were used to examine mechanisms for promoting iron uptake in *Zea mays*. Mutants included HM053, FP10, and *ipdC*, which have varying capacities for biological nitrogen fixation and production of the plant hormone auxin. Using radioactive iron-59 tracing and inductively coupled plasma mass spectrometry, we documented significant differences in host uptake of Fe^2+/3+^ correlating with mutant biological function. Radioactive carbon-11, administered to plants as ^11^CO_2_, provided insights into shifts in host usage of ‘new’ carbon resources in the presence of these beneficial microbes. Of the mutants examined, HM053 exhibited the greatest influence on host Fe uptake with increased plant allocation of ^11^C-resources to roots where they were transformed and exuded as ^11^C-acidic substrates to aid in Fe-chelation, and increased C-11 partitioning into citric acid, nicotianamine and histidine to aid in the in situ translocation of Fe once assimilated.

## Introduction

### The role of plant growth promoting bacteria in agriculture

A survey of the literature establishes that many plant growth promoting bacteria (PGPB) can strongly influence plant growth and increase crop yields. Mechanisms have been suggested that support these actions, which include: antagonism toward phytopathogens and induction of plant resistance pathways [1]; phytostimulation through microbial production and secretion of plant relevant hormones like auxin, cytokinins, and gibberellins, as well as nitric oxide [2, 3]; improvements in host nitrogen uptake *via* biological nitrogen fixation (BNF) [4]; and improvements in host micronutrient uptake [5, 6].

*Azospirillum brasilense*, a Gram-negative bacterium, is perhaps the best studied of the PGPB. It is known to be a prolific grass root colonizer preferring to grow on the outer surface of its host’s roots. As a diazotroph this bacteria has the ability to fix N_2_ and, in some circumstances, transfer that source of nitrogen to its host [4]. *A. brasilense* can also produce auxin which can affect plant development [3]. Over the years, commercial inoculants of various strains of this bacterium have been developed and tested in the field for their influence on growth performance in a variety of grain crops, including maize, under various environmental conditions [7–9]. In fact, recently we showed that maize inoculated with the functional mutant HM053 *A. brasilense* exhibited 12% larger stem diameters, 23% increased leaf thickness and 58% higher chlorophyll content relative to non-inoculated control plants when surveyed during 2018 and 2019 growing seasons [10]. Furthermore, plant growth promotion resulted in significantly higher crop yields compared to non-inoculated control plants where 34% and 53% increases were documented in the number of corn kernels per cob from inoculated plants during the 2018 and 2019 growing seasons, respectively. These phenotypic attributes were ascribed to increased host iron uptake in the presence of the beneficial microbe, which not only improved plant growth, but also had the effect of increasing seed iron content thus improving the nutritional value of the crop product.

### The importance of iron in plant growth

Iron is the third most limiting nutrient for healthy plant growth and development; however, this limitation is not attributable to its abundance. Quite the contrary, iron is the fourth most abundant element in the lithosphere. Even so, its bioavailability is limited especially in aerobic, neutral pH soils because it exists predominantly as insoluble Fe^3+^ oxyhydroxides that are unusable to plants [11, 12]. A deficiency of iron can decrease plant growth affecting crop yield and/or nutritional quality [13–15], while excess iron can lead to elevated Fe^3+^/Fe^2+^ redox reactions causing cellular damage.

Iron uptake in higher plants follows two mechanisms (Fig. S1), which are distinct between non-graminaceous plants and graminaceous plants. Non-graminaceous plants utilize a reduction-based strategy (*Strategy I*) through which protons are exuded from the roots *via* the actions of membrane-bound H^+^-ATPases [16–29]. These protons acidify the soil, solubilizing Fe^3+^. Additionally, membrane-bound Fe^3+^ reductase oxidases [20] reduce this solubilized iron to Fe^2+^ enabling its transport across the plasma membrane of the root epidermal cells through the actions of specialized ion transport proteins that are part of the larger ZIP (zinc-regulated transporter and iron-regulated transporter) encoding gene family [16, 17, 21–24]. Graminaceous plants like rice, barley and maize utilize a Fe^3+^ chelation mechanism (*Strategy II*) whereby phytosiderophores (PS) are excreted from the roots [25] where they chelate with Fe^3+^ in the soil. These PS-Fe^3+^ chelates are then taken into the root cells through either yellow stripe (YS) or yellow stripe-like (YSL) oligopeptide transporters first characterized in maize [26]. Even with these class distinctions nine ZIP-encoding genes have been identified in the maize genome with little known of their function, regarding Fe^2+^ uptake [24].

Once assimilated, iron will travel through the apoplastic space to reach the endodermis of the root. The endodermal ring can be a site rich in ion transporters facilitating the trafficking of several metal nutrients to the stele and xylem [27]. It also manifests as a natural barrier to ion trafficking in the form of the Casparian band and suberin lamellae. Here, a layer of waterproof lignin and suberin polymer exists, which can force ion nutrients to pass into the symplast [28]. Once in the vascular core, iron transport in the xylem occurs predominantly as the Fe^3+^–citrate complex [29–32]. However, iron is also capable of translocation in other forms including nicotianamine chelates (Fe-NA), mugineic acid chelates (Fe-MA) and dihydroxy mugineic acid chelates (Fe-DMA). NA is the direct amino acid precursor to all MAs and is produced from S-adenoysl methionine by nicotianamine synthase [33, 34]. Additionally, Fe^2+^ is capable of xylem translocation in the form of a histidine chelate [35].

### Beneficial microbes as the path forward to promoting plant growth

While evidence is compelling that certain beneficial microbes can influence plant growth through improved micronutrient uptake, we know little about the mechanisms of action, which, if fundamentally understood, could lay the foundation for translating this approach to general farming practices perhaps with greater acceptance by the public.

In the present work, we provide an in-depth analysis into the mechanisms of action for the beneficial effects of *A. brasilense* in maize. Our work relies on *A. brasilense* functional mutants providing a spectrum of biological functions spanning auxin biosynthesis to BNF. The use of functional mutants to examine plant-microbial interactions is not new to the field, and has been used extensively in other PGPB systems to examine the effects of associated microorganisms on plant physiology [36]. The functional mutants of *A. brasilense* used in the present study include HM053, a *Nif* ^+^ constitutively expressed strain hyper-fixing N_2_ and producing high levels of auxin; FP10, a *Nif* ^–^ strain deficient in N_2_-fixation, but still produces auxin albeit at a slightly lower capacity than HM053; and *ipdC*, a strain significantly inhibited in its capacity to produce auxin.

## Materials and methods

### Plant growth

Maize kernels from Elk Mound Seed Co. (Hybrid 8100) were dark germinated at room temperature for two days. Seeds were inoculated with bacteria culture as appropriate and transplanted to a growth pouch wetted with sterile Hoagland’s basal salt solution (PhytoTechnology Laboratories, Shawnee Mission, KS 66215, USA) for ~1 week. Seedlings were transferred to an aeroponics system (Fig. S2), and nutrients were replenished every five days. Two week old plants were used for all the ^59^Fe radiotracer and mass spectrometry studies.

For ^11^C physiology and metabolism studies, including root emission measurements, plants were grown in eight inch plastic cones filled with Turface™ (expanded clay matrix) where the bottom portion of the cone was immersed in de-ionized water (Fig. S3). Nutrient was introduced as Hoagland’s solution every three days. Growth conditions for both set-ups consisted of 12-h photoperiods, 500 μmol m^−2^ s^−1^ light intensity, and temperatures of 25 °C/ 20 °C (light/dark) with humidity at 70–80% for two weeks.

### Bacteria growth and root inoculation

Functional mutants of *A. brasilense* were obtained *via* a material transfer agreement between the corresponding author’s institution and the Federal University of Paraná (UFPR, Curitiba, PR CEP 81531-980, Brazil). HM053 originated as a natural mutant of the wild-type strain of *A. brasilense* FP2 (Sp7 ATCC 29145 Nif^+^ Sm^r^ Nal^r^) screened through its resistance to ethylenediamine (EDA^r^) according to the original work of Machado et al. [37, 38]. The FP10 mutant was obtained through the original work of Pedrosa and Yates [39] by N-nitrosoguanidine mutagenesis of the FP2 wild-type strain of *A. brasilense* and isolated by growth on glutamate medium. The indole-3-pyruvate decarboxylase gene (ipdC), coding for a key enzyme of the indole-3-pyruvic acid pathway of auxin (indole-3-acetic acid) biosynthesis in *A. brasilense*, was functionally disrupted in a site-specific manner using a SacB-cassette insertion into the ipdC gene of wild-type FP2 (Sp7 ATCC 29145) followed by homologous recombination. The method allowed for the construction of the *ipdC* mutation strain without unwanted sequence changes and relied on the λ Red recombineering (Direct and Inverted Repeat stimulated excision; DIRex) a method that works well for generating single point mutations, small insertions or replacements as well as deletions of any size, in a bacterial gene [40]. The resultant knock-out strain exhibited significant reduction in auxin biosynthesis to a level of 10% that of the wild-type strain [41].

Functional mutants were grown in liquid NFbHP-lactate medium following published procedures [4]. Cultures were washed with sterile water and diluted to ~10^6^–10^8^ colony forming units (CFU) per mL volume. Root inoculation involved adding 1 mL of inoculum to a Petri dish containing 10 maize seedlings (2 days after germination) and rocking in the shaking incubator for 2 h. Seeds were placed into plastic seed germination pouches (PhytoAB, Inc., San Jose, CA 95131, USA) for five days before transplanting to aeroponics or Turface™.

### Radioactive Fe-59 tracer studies

Two oxidation states of radioactive ^59^Fe (t_½_ 44.5 d) were used in the study, ferrous (Fe^2+^) and ferric (Fe^3+^), purchased from Perkin-Elmer Life Sciences (Akron, OH USA). One hour before administration of tracer, plants were removed from aeroponics growth cells and placed in 600-mL beakers with 100 mL of 5 μM Fe^3+^-EDTA to prohibit induction of water- or iron-stress responses during the study. In auxin treatment studies, 50 mL of 30 μM indole-3-acetic acid (auxin) was added to 50 mL of the iron solution for a total of 100 mL. Iron radiotracer of 0.74 MBq was introduced. After 3 h, the roots were cut from the shoots and washed in water followed by an EDTA solution, then blotted dry. The root and shoot tissues were counted in a three inch NaI (PMT: Photomultiplier Tube) gamma well-type detector for percent assimilation and allocation calculations.

### Production and administration of radioactive ^11^CO_2_

^11^CO_2_ (t_½_ 20.4 min) was produced on the GE PETrace Cyclotron located at the Missouri Research Reactor Center using high-pressure research grade N_2_ gas target irradiated with a 16.4 MeV proton beam to generate ^11^C *via* the ^14^N(p,α)^11^C nuclear transformation [42, 43] using our published procedures where details can be found in the Supplementary Methods [44].

### Whole-plant ^11^C-physiology measurements

After pulse labeling using ^11^CO_2_, plants were incubated for 3 h before separating the load leaf, shoots, roots and growth media. Roots were washed in 100 mL de-ionized water to remove surface root exudates. Measurement of ^11^C-activity was performed using gamma counting and data was decay corrected to end-of-bombardment. The individual components (plant tissues, growth media and root wash) were summed for total plant ^11^C-activity. Individual components were used to calculate leaf export, root allocation and root exudation fractions. Aliquots of the root washings were processed through a 1 mL column packed using 200 mesh AG1 X-8 anion exchange resin (Bio-Rad Laboratories, Inc., Hercules, CA, USA). Afterward, the column and breakthrough rinse were counted for radioactivity providing a measure of the acidic and non-acidic fractions of root exudates.

### [^11/12^C]metabolite analyses

In separate studies, leaves were exposed to ^11^CO_2_ for 20 min before metabolite analyses following published procedures [45]. Tissues were flash frozen in liquid nitrogen, ground to a fine powder and extracted in methanol: water (60:40 v/v) in Eppendorf™ tubes. After centrifugation the insoluble and soluble portions were separated and counted for ^11^C-activity using a NaI (PMT) gamma counter. The insoluble portion contained mostly cell-wall polymers and starch. The soluble portion contained small water-soluble compounds, including sugars, amino acids and non-nitrogen containing organic acids. All data was decay corrected back to the end-of-bombardment. [^11^C]-Sugars were analyzed by radio thin layer chromatography (TLC) using glass backed NH_2_-silica HPTLC-plates (200 µm, w/UV254) purchased from Sorbent Technologies (Atlanta, GA, 30071, USA) according to published procedures [46, 47] (see Supplementary Methods).

[^11^C]Amino acids were analyzed following published procedures [45] using pre-column OPA derivatization and quantified by gradient radio HPLC (see Supplementary Methods).

For [^11^C]organic acid analysis, leaf extract was processed through a QMA Sep-Pak™ then rinsed with 10 mL of deionized (DI) water followed by 1 mL of 20 mM sodium phosphate buffer (pH = 1.5). Aliquots of the phosphate rinse were analyzed using a gradient radio HPLC equipped with a UV detector (210 nm) and an on-line NaI (PMT) radiation detector. The acids were separated using a Phenomenex Gemini 5 µm C18 (250 × 4.6 mm inner diameter) column with a solvent system of solvent A (20 mM potassium phosphate monobasic pH of 2.5) and solvent B (60:40 methanol: acetonitrile: (v/v)) starting at 100% A at injection and ramping to 75:25 A:B within 10 min at a flow rate of 1 mL min^−1^. Metabolite mass and radioactivity were recorded and quantified as described above for amino acids.

[^11^C]Nicotianamine was analyzed by reacting a portion of the QMA extract with 10 mM ferrous sulfate heptahydrate (1:1 v/v) for 30 min to chelate the nicotianamine. A radio HPLC system was used in the analysis (*see* Supplementary Methods) where 20 mM potassium phosphate buffer at pH 2.50 (solvent A) and 60:40 methanol:acetonitrile (solvent B) were pumped through a Phenomenex Gemini C18 110 A 250 × 4.60 mm column. The solvent gradient changed from 100% solvent A to 75%:25% A:B at 10 min, and back to 100%A by 20 min where it was held for 5 min. The chelated sample was measured by UV absorption at 243 nm. The amount of nicotianamine mass per gram fresh weight tissue was assessed for control, HM053, FP10 and *ipdC* treatments using authentic nicotianamine standards to calibrate the detector response. Radioactivity associated with this metabolite was measured by NaI (PMT) detector connected in series with the UV detector.

Specific activities (SA) of individual metabolites of interest including [^11^C]histidine, [^11^C]citric acid and [^11^C]nicotianamine were calculated using Eq. (1) following procedures described in the Supplementary Methods:

1 $${\mathrm{SA}} = \! \left( {\% \,{\mathrm{Fixed}}^{11}{\mathrm{C}}\,{\mathrm{Activity}}} \right)/(\mu {\mathrm{moles}}\,{\mathrm{of}}\,{\mathrm{metabolite}}\,{\mathrm{mass}} \times {\mathrm{gfw}}^{ - 1}{\mathrm{tissue}})$$

### [2-^11^C]indole and its application to measuring microbial auxin biosynthesis

Radiosynthesis of [2-^11^C]indole followed published procedures using [^11^C]hydrogen cyanide [48]. Synthesis took less than 1 h providing product at >98% radiochemical purity, and specific activity of 176 ± 24.8 GBq μmol^−1^.

Aliquots of purified radiotracer were dispensed into 10 mL Falcon tubes containing thoroughly washed and harvested bacteria re-suspended in sterile water. Bacteria count was measured by sample turbidity, where OD_600_ = 1.0 (Optical Density at 600 nm, corresponded to 10^8^ cells mL^−1^). Once tracer was introduced, tubes were gently rocked at ambient temperature for up to 2 h and samples were periodically removed for analysis of [2-^11^C]indole-3-acetic acid conversion.

[2-^11^C]indole-3-acetic acid analysis was carried out using radio HPLC using a Phenomenex Gemini 5 µm C18 (250 × 4.6 mm inner diameter) column at 35 °C and a mobile phase composed of acetonitrile:water (50:50, v/v %) at flow rate of 1.0 mL min^−1^. The fluorescence detector was set with excitation and emission wavelengths of 230 nm and 360 nm, respectively with a NaI (PMT) gamma radiation detector in series.

### Inductively coupled plasma-mass spectrometry (ICP-MS)

For ICP-MS analyses, roots and shoots were dried in an oven, weighed into digestion vessels and digested in 3.0 mL of concentrated nitric acid at 190 °C in a Milestone Ethos Plus (Milestone SRL, Sorisole, Italy) microwave digestion system. Digestants were diluted to 50 mL with ultrapure water and gravimetrically diluted by a factor of 10 with 0.45 N nitric acid. Samples were analyzed *via* Perkin-Elmer NexION ICP-MS in Kinetic Energy Discrimination mode. Total elemental ion counts were measured of ^56^Fe and normalized to ^12^C counts. Reference materials included NIST SRM 1570 spinach leaves and NIST SRM 1573 tomato leaves prepared as samples were. Internal Calibration standards of Sc, In, and Tl at known concentrations were used from stock solutions (High Purity Standards, Charleston, SC 29418, USA).

For Laser Ablation-ICP-MS, roots were removed from shoots and a 3-cm section of primary lateral root was further excised above the apical meristem. Roots were sectioned in OCT embedding media to 100 μm thickness (Fisher Scientific Inc., Hampton, NH 03801, USA) and placed on quartz microscope slides for freeze drying in a FreezeZone 1 dryer (Labconco Corp., Kansas City, MO, USA) before ablation.

### Transmission electron microscopy

Specimens were collected from control and inoculated groups and processed for transmission electron microscopy (TEM) using standard procedures of the U. Missouri Microscopy Core, details in the Supplementary Methods.

### Root ethylene emission measurements

Aeroponically grown plants were harvested at two weeks, roots weighed and placed in air-tight jars equipped with a sampling port. Roots from 2–3 plants were binned generating a single data point. Roots volatilized for 90–130 h and 5-mL of head space was sampled using a gas-tight syringe. The sample was injected into a flame-ionization Hewlett Packard 5890 A gas chromatograph with a 2-m long, 1-mm inner diameter ShinCarbon ST packed column. The program started with a 2 min hold at 40 °C and increased 10 °C min^−1^ to 250 °C. The injector temperature was 250 °C and the flame ionization detector temperature was 300 °C. Chromatographic peaks for ethylene were measured using PeakSimple™ chromatography software and quantified against ethylene standards. Ethylene emission values were reported as ρmol ethylene gfw^−1^ tissue hr^−1^ as a function of bacteria treatment.

### Root gravitropism measurements

Maize seeds were placed upright in Gelrite™ solid media in a growth box. The box was rotated 90° after root growth reached half way down the box in the Gelrite™ media. Photographs of the roots were taken after 12 h. Root bending angles were measured with Image-J software. The degree of bending as a function of bacterial treatment was assessed as an indirect measurement of plant-root auxin status.

### Root indole measurements

Fresh roots were harvested from two week old plants, weighed, and placed in air-tight jars equipped with two sampling ports. The roots volatilized for 48–90 h. A glass tube trap was loaded with 50 mg Tenax® GR, a composite mix of Tenax® TA (Scientific Instrument Services, Inc., NJ 08551, USA), porous polymer based on 2, 6-diphenylphenol with 30% graphite additive. This trap was affixed to one port equipped with a silicon rubber seal and an aquarium air pump was affixed to the other port. Air was pumped through the chamber for 1 h at a rate of 50 mL min^−1^ providing three complete air exchanges. Tenax® traps were rinsed with 0.5 mL of methylene chloride and 1–3 μL volumes of this extract were analyzed by capillary gas chromatography using a Hewlett Packard 5890 A gas chromatograph equipped with a 30-m long, 0.53 mm inner diameter, 0.25 µm film thickness, Restek RTX-WAX column for separation and a flame ionization detector. The program was as described for ethylene measurements. Chromatographic peaks for indole eluted at 15 min and were measured using PeakSimple chromatography software and quantified against standards. Root indole volatile emission rates were reported as ρmole indole gfw^−1^ h^−1^ as a function of bacteria treatment.

### Root DIMBOA measurements

Maize root tissue (700–800 mg) was macerated by mortar and pestle in 2.00 mL of DI water. The resulting extract was centrifuged, combined with 1 mL of ethyl acetate (ETAC) and gently rocked for 1 min. The DIMBOA/ETAC organic layer was removed and the aqueous phase was re-extracted with 1 mL ETAC and combined with the first organic sample. An aliquot of the organic phase was injected into a Hewlett Packard 5890 A gas chromatograph equipped with a 30-m long, 0.53 mm inner diameter, 0.25 µm film thickness, Restek RTX-WAX capillary column and flame ionization detector. The program started with a 2 min hold at 70 °C and increased 10 °C min^−1^ to 250 °C. The injector temperature was 250 °C and the flame ionization detector temperature was 300 °C. The retention time of DIMBOA was ~24 min. Chromatographic peaks for DIMBOA were measured using PeakSimple chromatography software and quantified against authentic standards (Santa Cruz Biotechnology, Inc., TX, USA). Root DIMBOA values were reported as μmole DIMBOA gfw^−1^ tissue as a function of bacteria treatment.

### In vitro chemotaxis assays to examine effects of DIMBOA on bacteria growth

A standard of DIMBOA was prepared by dissolving 10 mg of compound in 6 mL of deionized sterile water using 200 μL DMSO (3% v/v) to facilitate sample dissolution. Aliquots of this standard were then added to bacteria growth media (as described in *Bacteria Growth Methodology* section) to generate 0.02 mM, 0.1 mM and 0.5 mM concentrations of DIMBOA. Each dose was replicated in triplicate. The amount of DMSO for each dose was adjusted to match that of the highest DIMBOA concentration. Additionally, 0 mM DIMBOA controls were prepared (also in triplicate) where an equivalent amount of DMSO as that found in the highest 0.5 mM DIMBOA dose was introduced to null effects of this organic solvent on bacteria growth. *A. brasilense* cultures of the functional mutants HM053, *ipdC* and FP10 were introduced into these modified media and grown under previously described conditions for 24 h after administration of DIMBOA to the growth media. An additional sample of media containing 0.5 mM DIMBOA, but no bacteria, was set aside for examining DIMBOA stability at 30 °C. OD_600_ measurements were taken using a ThermoScientific Evolution 201 UV-vis spectrophotometer at 24 h into the bacteria growth cycle. The 0.5 mM DIMBOA cultures were re-measured after 48 h of incubation. DIMBOA stability measurements were conducted over 27 h using capillary gas chromatography and flame ionization detection using the analytical methodology previously described above. Samples were prepared for GC injection by extracting 200 μL of growth media using 500 μL of ETAC. Aliquots of the organic extract were then injected into the gas chromatograph using a 1:20 sample split upon injection.

### Statistical analysis

Data was subjected to the Shapiro–Wilk Normality Test to identify outliers so all data groups reflected normal distributions. Data was analyzed using the Student’s t-test for pair-wise comparisons made between non-inoculated controls and bacteria treatment. Statistical significance was set at *P* < 0.05. The ^59^Fe^3+/2+^ allocation data was analyzed by Principal Component Analysis (PCA) using XLSTAT software version 2020.3 (Addinsoft Inc., New York, NY 10001, USA).

## Results and discussion

### Bacteria influence host uptake and translocation of iron

**Fig. 1:**
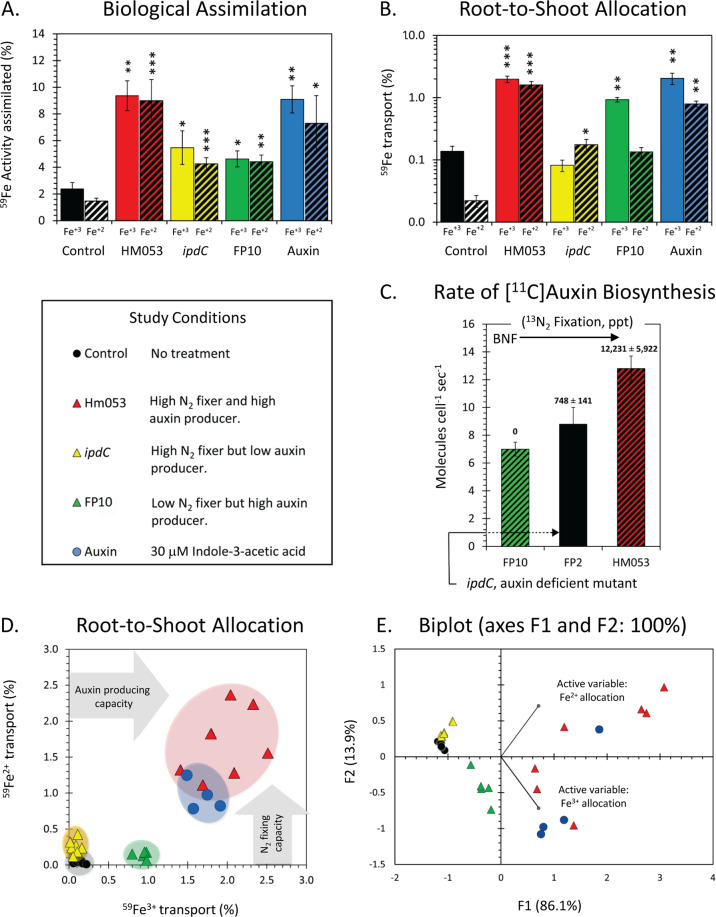
Radioactive Fe-59 reveals features for *A. brasilense* promotion of host assimilation and whole-plant transport of ferrous iron (Fe^2+^) and ferric iron (Fe^3+^). **A** Bar graph of biological assimilation of radioactive ferrous ^59^Fe^2+^ and ferric ^59^Fe^3+^ after 3 h of incubation of maize roots using 0.74 MBq of tracer. Biological assimilation reflects a combination of root and microorganism assimilation of tracer. Data presented as weight normalized percentage of ^59^Fe radioactivity administered. Asterisks indicate significant differences of treatment relative to control (*P < 0.05; **P < 0.01; ***P < 0.001). **B** Bar graph showing the extent of root-to-shoot transport of radioactive ^59^Fe^2+^ and ^59^Fe^3+^ after 3 h of incubation of maize roots using 0.74 MBq of tracer for the same five study conditions. Data presented as weight normalized percentage of the assimilated ^59^Fe radioactivity. Asterisks indicate significant differences of treatment relative to control (*P < 0.05; **P < 0.01; ***P < 0.001). **C** Bar graph showing auxin biosynthetic rates in *A. brasilense* using [^11^C]indole radiotracer reveals a trend of increasing rate of production with microbial BNF capacity. Correlations were made to BNF data measured using ^13^NN [4]. Data (±SE) reflects *N* = 4 biological replicates. **D** Correlation plot mapping ^59^Fe^2+^ allocation against ^59^Fe^3+^ allocation for the study conditions reveals systematic trends of clustering with treatments that correlate to auxin producing and N_2_-fixing capacities of the beneficial microbes. **E** Principal component analysis correlates ^59^Fe translocation to biological functions of the beneficial microbes and to chemical treatment using auxin.

**Fig. 2:**
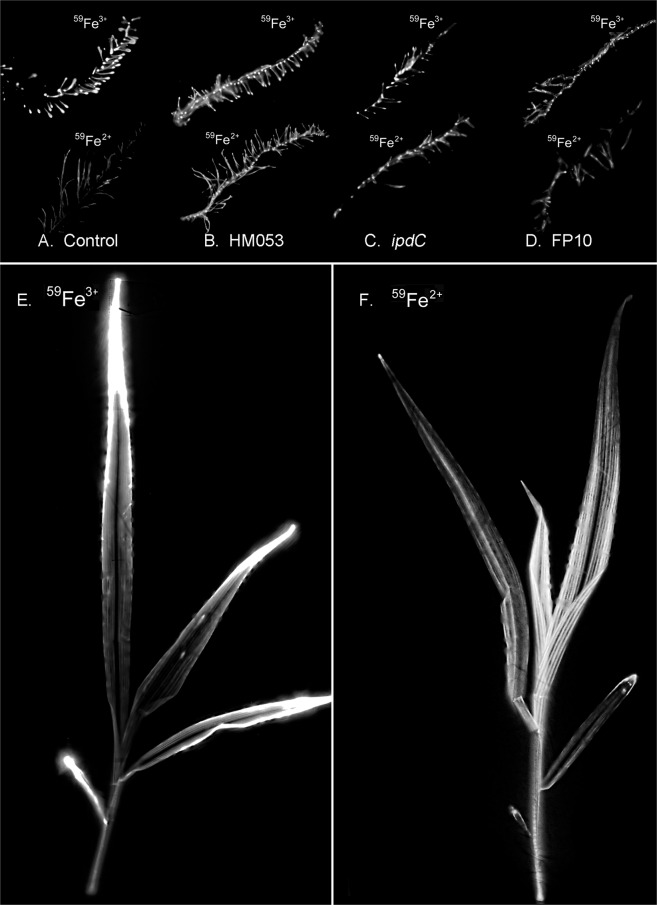
Radiographic imaging of plant tissues reveal different spatial patterning of radioactive ^59^Fe as ferric (Fe^3+^) and ferrous (Fe^2+^) ionic forms. Comparisons were made between non-inoculated control roots (**A**) and roots inoculated with HM053 (**B**), *ipdC* (**C**) and FP10 (**D**) strains of *A. brasilense* with ^59^Fe^3+^ images are shown in the upper portion of each panel while ^59^Fe^2+^ images are shown in the lower portion. ‘Hot’ spots of ^59^Fe radioactivity (both for ^59^Fe^3+^ and ^59^Fe^2+^) are seen at lateral root junctions. Roots inoculated with the FP10 strain of *A. brasilense* only showed ^59^Fe^3+^ ‘hot’ spots. Shoot images for HM053 inoculated plants are shown for ^59^Fe^3+^ (**E**) and ^59^Fe^2+^ (**F**). Translocation of ^59^Fe^3+^ to shoots resulted in higher tracer accumulation in all of the leaf tips. Translocation of ^59^Fe^2+^ to shoots resulted in a higher distribution of tracer in the younger developing leaf tissue.

Radioactive ^59^Fe^3+^ and ^59^Fe^2+^ tracers were applied to roots inoculated with the three strains (HM053, FP10 and *ipdC*) of *A. brasilense*. Comparisons in host iron assimilation were made to non-inoculated control plants and to plants chemically treated with auxin. As expected from the *Strategy II* iron uptake mechanism of grasses, non-inoculated maize roots showed higher ^59^Fe^3+^ uptake than ^59^Fe^2+^ (Fig. 1A). Qualitatively, radiographic imaging following tracer administration in non-inoculated control plants revealed high uptake of ^59^Fe^3+^ in root meristems and lateral roots (Fig. 2A). ^59^Fe^2+^ was taken up to a much lesser extent. Quantitative tissue counting and root radiographic images of roots inoculated with the functional mutants of *A. brasilense* revealed very different behavior. The effects of bacteria on host iron assimilation (Fig. 1A) and root-to-shoot translocation (Fig. 1B) are striking. Especially with regard to the HM053 functional mutant, such effects on the host plant seem to be tied to biological nitrogen fixing (BNF) and auxin producing capacity of the microorganism. Drop plate assays examining the degree of microbial colonization under our plant growth conditions (Fig. S4) provided evidence that inoculation with all three functional mutants generated between 6.3 × 10^6^ and 3.2 × 10^7^ CFUs gfw^−1^ of root tissue relative to roots from non-inoculated control plants yielding a substantially lower background microbial count of 6.3 × 10^5^ CFUs gfw^−1^. HM053 showed the greatest degree of colonization which could account, in part, for its enhanced performance. However, *ipdC* and FP10 exhibited the same levels of colonization, yet FP10 outperformed *ipdC* in influencing host iron assimilation. The extent of colonization does not fully explain the actions here, and thus microbial biological functions must play a role.

The spatial patterning of Fe-59 radioactivity in roots associated with bacteria was different from non-inoculated control roots, with increased ^59^Fe^3+^ and ^59^Fe^2+^ accumulation noted at the lateral root junctions (Fig. 2B–D). FP10 was the exception, which did not exhibit significant ^59^Fe^2+^ assimilation, but substantial ^59^Fe^3+^ assimilation (Fig. 2D). The spatial patterning of radioactivity is consistent with published confocal microscopy images demonstrating the lateral root junctions typically hold high populations of these bacteria [4]. Differences in spatial patterning exhibited by non-inoculated and inoculated roots suggests bacteria assimilation of iron can contribute to a significant portion of the measured root radioactivity detected in the NaI (PMT) gamma counter even after thorough root washing.

Systematic trends defining *in planta* translocation (Fig. 2E, F) of iron become apparent in the correlation plot (Fig. 1D) of ^59^Fe^2+^ transport versus ^59^Fe^3+^ transport and in the PCA biplot (Fig. 1E). Here, the information included in our allocation measurements were represented by feature vectors (F1 and F2) representing 86.1% and 13.9% of the information embedded in the data respectively (for more detail see Supplementary Information). As displayed, each of the treatments clustered together, indicating behavior within a treatment-type that is distinct from other treatments. It shows that *ipdC* and non-inoculated maize are similar in overall iron allocation behavior, while auxin chemical treatment and HM053 inoculated maize share similar allocation patterns. FP10 is most unique in its allocation patterns. Non-inoculated maize and *ipdC* inoculated maize clustered away from active variable vectors. This indicates both treatment types do not allocate much iron overall compared to other treatments, and *ipdC* narrowly allocates more ^59^Fe^2+^ than ^59^Fe^3+^. FP10 inoculated maize are distinctly apart from other treatments in the negative X- and Y-axis direction. This is more positively correlated with Fe^3+^ allocation and negatively with Fe^2+^ allocation - which is observed in the correlation plot (Fig. 1D), as well. Finally, the auxin chemical treatment and HM053 inoculated maize share positions along the active variable vectors, indicating they have the largest allocation percentages across all treatment types for both ^59^Fe^3+^ and ^59^Fe^2+^. HM053 inoculated maize tend to have the largest allocation values, especially for the ^59^Fe^2+^ as noted by the majority of points along that active variable vector while the auxin chemical treatment tends to show more ^59^Fe^3+^ than ^59^Fe^2+^ allocation as indicated by its position on the biplot.

To summarize, allocation of ^59^Fe^2+^ appeared stimulated by BNF while both ^59^Fe^3+^ and ^59^Fe^2+^ appeared stimulated by the auxin biosynthetic capacity of the inoculated bacterium. This assumes bacteria-borne auxin or indole-like substrates move from the microorganism into their host. Exogenously applied chemical auxin stimulated host iron assimilation and whole-plant iron translocation just as HM053 inoculated plants did (Fig. 1A, B). BNF and auxin biosynthesis are not mutually exclusive microbial functions. BNF capacity seems to impact bacterial ability to produce auxin (Fig. 1C), although it is not essential for that function. FP10, the BNF deficient strain, was able to biosynthesize auxin at a rate of 7.0 ± 0.4 molecules cell^−1^ s^−1^, slightly less than the 9.2 ± 0.9 molecules cell^−1^ s^−1^ production rate demonstrated by the FP2 wild-type strain and significantly less than the 13.4 ± 0.9 molecules cell^−1^ s^−1^ auxin production rate of the HM053 strain. Though not directly measured by our group using tracer analysis as done with other mutants, *ipdC*’s capacity to produce auxin was reported to be ~10% that of the wild-type strain [41].

### Bacteria influence host cellular-scale iron distribution

**Fig. 3:**
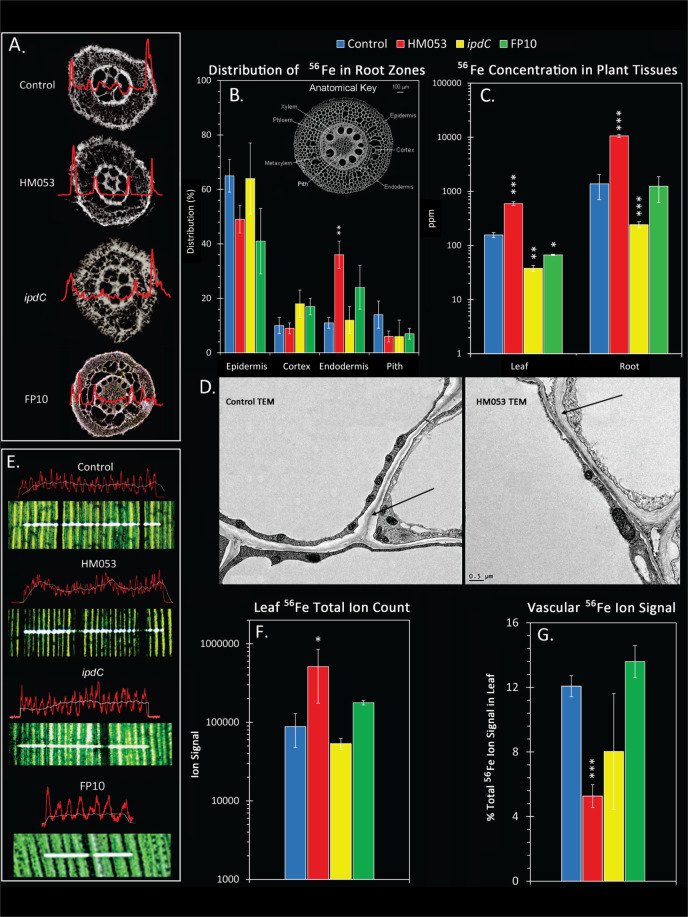
LA-ICP-MS and TEM reveal spatial patterning of Fe-56 in plant tissues that correlates with root cellular morphological changes due to beneficial microbes. **A** Representative Fe-56 ion signals (in red) from the laser ablation of 100 μm root sections (taken 1 cm from the root tip) from non-inoculated control plants, and plants inoculated using HM053, *ipdC* and FP10 bacteria. **B** Data from ablation tracks was averaged across *N* = 6 biological replicates, and presented graphically as % distribution of the integrated Fe-56 ion signal for the different regions of the root cross section (epidermis, cortex, endodermis and inner pith region). These regions are identified on the inset anatomical key. HM053 treatment resulted in significantly higher iron levels in the endodermis than controls. **C** Absolute concentrations of Fe-56 (ppm) in leaves and roots were determined from direct injection ICP-MS of digested tissues and compared against NIST standards. **D** Transmission electron microscopy images on endodermal root cells reveal morphological differences between cell walls of non-inoculated control roots and those from HM053. HM053 inoculated roots appeared to have a more defined Casparian band in endodermal radial root walls than in control plants (highlighted by black arrows). **E** Representative Fe-56 signals from 40 μm diameter x 2 mm laser tracks rastered across freeze dried leaf tissues harvested from non-inoculated, HM053, *ipdC* and FP10 inoculated plants. The periodic patterning of the iron ion signals (red lines) presumably are due to the higher disposition of iron in leaf cell walls after the freeze drying process. The white lines reflect an average ion signal across the laser track. HM053 elicits a unique patterning with lower iron signal in and around vascular tissues that was not seen with the other beneficial microbes. **F** The ion signal for Fe-56 was integrated across the laser ablation track and plotted as a function of biologic type. HM053 showed a higher integrated iron signal than non-inoculated control, as well as *ipdC* and FP10 inoculated plants. **G** Relative percent of the Fe-56 ion signal in vascular tissue was plotted as a function of biologic type. HM053 inoculation decreased the relative percentage of iron in leaf vascular tissue suggesting greater mobility. Data (±SE) reflected *N* = 4–6 biological replicates. Asterisks indicate significant differences of treatment relative to control (*P < 0.05; **P < 0.01; ***P < 0.001).

Laser ablation-inductively coupled-mass spectrometry (LA-ICP-MS) was applied to root sections to corroborate radiotracer data by spatially mapping stable Fe-56 at cellular scales. Significantly, elevated Fe-56 accumulation was observed in the endodermal ring from HM053, but not *ipdC* or FP10 inoculated plants, nor in non-inoculated control root sections (Fig. 3A, B). Digestive ICP-MS analysis enabling measurement of absolute Fe-56 concentrations in roots and leaves (Fig. 3C) showed that HM053 significantly increased whole-plant tissue iron levels 4-fold in leaves and 10-fold in roots relative to controls, corroborating radiotracer experiments. Plants inoculated with *ipdC* showed lower levels of Fe-56 than non-inoculated controls. Though not entirely consistent with our radiotracer data, we note that levels of Fe-59 allocation over the short term were very small such that over a longer period of plant growth, iron accumulation could manifest in the same or lower iron signature than controls. Similarly, FP10 showed only slightly lower or same levels of iron as controls.

To investigate high iron accumulation in the endodermal ring of HM053 inoculated plants, we used transmission electron microscopy to examine the radial walls of root endodermal cells (Fig. 3D). Morphological differences were seen between non-inoculated and HM053-inoculated roots with HM053 showing more prominent development of the Casparian band between cell walls. The Casparian band is a layer of waterproof lignin and suberin located at the endodermal ring. This barrier forces iron nutrients to pass from the apoplast into the symplast as they move to the stele and vascular core [27]. Once iron has entered the symplast, it is bound to various chelators that facilitate its transport as Fe^3+^-chelate. However, the endodermal ring can be a site harboring a rich density of metal transporters that may transport Fe^2+^ as well as other metals [27].

High accumulation of iron in the endodermal ring seen with HM053 inoculation implies the bacteria must influence cellular transport of this element, whether as Fe^2+^ or Fe^3+^, from the outer epidermal cells to the endodermal ring. Because our LA-ICP-MS analyses were conducted on freeze-dried root sections, likely metal ion distributions across each cell will be affected. Therefore, we are reluctant to draw any conclusions at this time on whether apoplastic transport dominated iron cellular trafficking. In future studies, we hope to re-examine this behavior once a cryostage is constructed enabling laser ablation of fresh tissue sections.

There is evidence, however, that cellular iron transport is promoted by HM053 from leaf tissue analyses using LA-ICP-MS (Fig. 3E–G). Here, HM053 inoculated plants significantly increased Fe-56 transport out of foliar vascular tissues (Fig. 3G) relative to controls while *ipdC* and FP10 inoculated plants showed no difference in vascular tissue iron levels compared to controls.

### Bacteria influence host use of its carbon resources

**Fig. 4:**
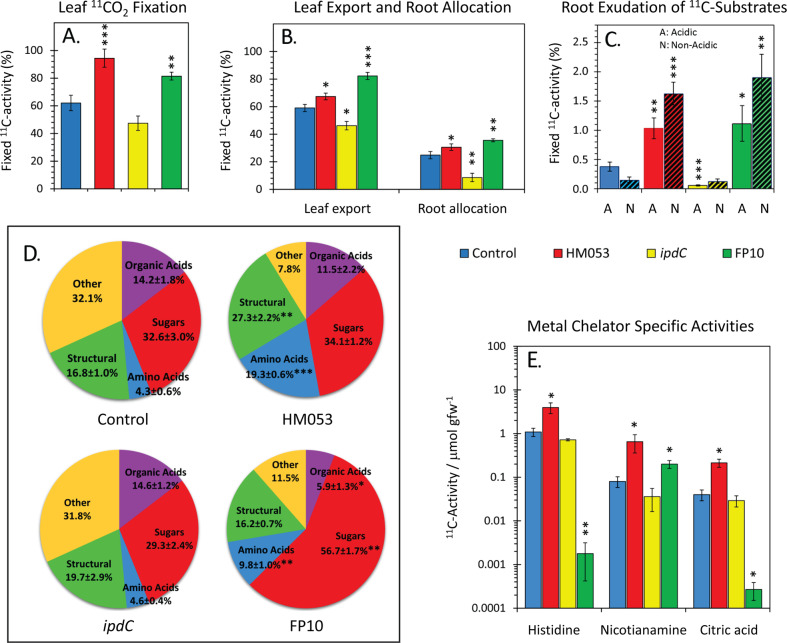
Carbon-11 aids in mapping maize physiological and metabolic responses to bacteria inoculation. **A** Leaf tissue was normalized to ^11^CO_2_ fixation and presented as % ^11^C-activity in the pulse applied to the leaf cuvette. **B** Leaf export and root allocation of ^11^C-photosynthates (measured at 3 h post ^11^CO_2_ pulse) presented as % fixed ^11^C-activity by the plant. **C** Root exudation of ^11^C-substrates (A = acidic substrates; N = non-acidic substrates measured at 3 h post ^11^CO_2_ pulse) presented as % fixed ^11^C-activity by the plant. **D** Metabolic landscape reflecting the partitioning of ‘new’ carbon (as ^11^C) into different metabolic pools of the load leaf tissue. **E** [^11/12^C]-histidine, [^11/12^C]-nicotianamine and [^11/12^C]-citric acid specific activities (SA) levels measured in leaf tissue 20 min. after tracer administration and represented as % total ^11^C-activity/μmol substrate gfw^−1^ of tissue. Data (±SE) reflects N = 7–9 biological replicates. Asterisks indicate significant differences relative to control (*P < 0.05; **P < 0.01; ***P < 0.001).

The effect of HM053, *ipdC* and FP10 inoculants on their host’s physiological and metabolic posture was examined to better understand the mechanism for bacterial induced enrichment of plant iron. Administration of radioactive ^11^C to live plants as ^11^CO_2_ allowed the measurement of leaf carbon fixation, export of ^11^C-photosynthates from shoots-to-roots, and exudation of ^11^C-substrates to the surrounding rhizosphere (Fig. 4A–C). These experiments showed that the functional mutants induced significantly different host physiological responses relative to non-inoculated control plants and each other. Increased carbon input *via* ^11^CO_2_ fixation with HM053 and FP10 inoculants correlated with higher root allocation of carbon resources and root exudation (Fig. 4A–C). Exudation of acidic substrates with these functional mutants was 2.8-fold and 2.1-fold higher for HM053 and FP10 inoculated plants, respectively, than non-inoculated control plants (Fig. 4C). This behavior was confirmed with a pH visual assay (Fig. S5). Taken together, these observations could help explain the noted increase in *Strategy II* iron assimilation using HM053 and FP10 inoculants because certain acidic exudates behave as chelating agents of Fe^3+^. Additionally, different patterns of metabolic partitioning of ‘new’ carbon (as ^11^C) into the plant’s metabolic landscape were noted between the functional mutants and non-inoculated control plants (Fig. 4D). In particular, HM053 and FP10 inoculation significantly increased ^11^C partitioning into the amino acid (AA) pool 4.5-fold and 2.3-fold, respectively, relative to controls. Inoculation with *ipdC* did not have an effect on the AA pool relative to controls.

Other subtle differences in the metabolic landscape were seen including HM053 influence increasing host ‘new’ carbon partitioning into structural metabolite pools (Fig. 4D). This strongly correlates with our prior work demonstrating that plants inoculated with HM053 had thicker leaves and larger stems than non-inoculated controls [10].

Insight into particular points of metabolic regulation and the bearing these actions may have on host iron transport prompted examination of individual metabolite ^11/12^C specific activities (SA). Large changes in metabolite SA values reflect high metabolic input of ‘new’ carbon resources while substrate endogenous concentrations are being depleted by the host’s demands for a particular metabolite. SA values for [^11/12^C]histidine, [^11/12^C]nicotianamine, and [^11/12^C]citric acid were significantly elevated with HM053 inoculation relative to control plants. Plants inoculated with FP10 showed significant elevation in [^11/12^C]nicotianamine SA, but significantly lower values for [^11/12^C]histidine and [^11/12^C]citric acid relative to controls. *ipdC* inoculation had no effect on SA values (Fig. 4E).

Histidine, one of the least abundant AAs in plant metabolism, plays important roles as a proton shuttle substrate in the electron transport train of the plant’s photosynthetic PSII system [49]. HM053 promoted increased ^11^CO_2_ fixation (Fig. 4A) suggesting up-regulation of leaf photosynthesis. However, this may also be due in part to observed increases in leaf chlorophyll with HM053 inoculation [10].

We note that histidine is also an important chelating agent in the cell-to-cell transport of divalent metals including Fe^2+^ [50]. Hence, the significant increase in [^11/12^C]histidine SA with HM053 inoculation may, in part, contribute to the increased translocation of ^59^Fe^2+^ (Fig. 1B). Inoculations with *ipdC* or FP10 bacteria had no effect or decreased [^11/12^C]histidine SAs relative to controls, respectively. Recall that *ipdC* showed slightly elevated levels of ^59^Fe^2+^ allocation relative to controls while FP10 showed a significant reduction in ^59^Fe^2+^ allocation.

Like histidine, nicotianamine and citric acid are also important chelating agents of iron, facilitating transport through the plant’s vascular architecture [51]. Both HM053 and FP10 inoculates significantly elevated [^11/12^C]nicotianamine SAs while only HM053 increased [^11/12^C]citric acid SA. Together, these changes in host metabolic regulation are relevant to explaining why HM053 was the better performer in stimulating host iron assimilation and allocation.

### Bacteria-borne indole could influence host root function

**Fig. 5:**
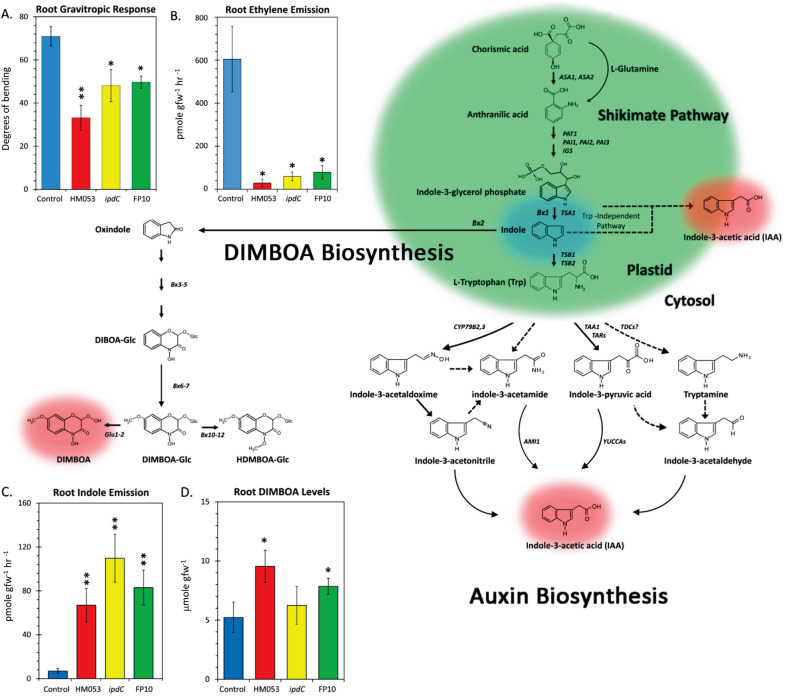
Shared metabolic branch point in the biosynthesis of Auxin and DIMBOA. The biosynthesis of auxin precursors such as indole-3-glycerol phosphate, indole and L-Trp takes place in plastids and are generated *via* the shikimate pathway. Four putative L-Trp pathways for auxin biosynthesis are shown. Enzymes known to operate these pathways are shown in italics. Solid pathway arrows reflect active processes. Dashed arrows are suggested to exist, but have not been proven. Benzoxazinoid biosynthesis yielding DIMBOA and its conjugate analogs share a common metabolic branch point with auxin as indole. **A** Gravitropic responses of root tips are associated with auxin biosynthesis and/or sensing. Inoculation of roots using HM053, *ipdC* and FP10 bacteria reduced root gravitropic responses relative to controls. **B** Auxin and ethylene biosynthesis are synergistic – inoculation of roots using HM053, *ipdC* and FP10 bacteria reduced root ethylene emission rates relative to non-inoculated controls. **C** Root indole emission rates were significantly elevated relative to controls in roots inoculated with HM053, *ipdC* and FP10 bacteria. **D** Root DIMBOA concentrations were significantly increased relative to control in roots inoculated with HM053 and FP10 bacteria and unchanged for *ipdC* bacteria. Data (±SE) reflects N = 6–8 biological replicates. Asterisks indicate significant differences relative to control (*P < 0.05; **P < 0.01; ***P < 0.001).

Additional influences of *A. brasilense* on host root performance were examined to better understand the mechanisms for iron enrichment. Ethylene, auxin, DIMBOA, and indole are interrelated in regulation and/or root metabolic and physiological functions. DIMBOA, a benzoxazinoid secondary metabolite in maize, shares a common branch point with auxin biosynthesis from the shikimate pathway at the indole precursor (Fig. 5). Therefore, the bacteria’s own auxin producing capabilities might influence host root auxin biosynthesis and/or sensing.

In all higher plants, indole is produced from indole-3-glycerol phosphate and channeled into different pathways. Indole is transformed into tryptophan, and later auxin, by the tryptophan synthase-α subunit and tryptophan synthase-β subunit [52, 53]. In maize specifically, indole is also produced by the BX1 enzyme as an intermediate in the production of DIMBOA, and other benzoxazinoid secondary metabolites, which play important roles as allelochemicals in maize defense [54, 55]. However, indole and its role in maize metabolism is more complicated than serving as a metabolic intermediate for DIMBOA or auxin biosynthesis. Aside from the previously mentioned routes, it can also be formed by indole-3-glycerol phosphate lyase (IGL), which subsequently releases it as a volatile in response to herbivore attack [56]. Indole release has been shown to occur earlier than other induced biochemical responses where the *Igl* gene is induced in response to the actions of certain fatty acid derivatives such as ***N****-*(17-hydroxylinolenoyl)-L-glutamine isolated from regurgitate of some herbivore-pests [57]. Hence, as a chemical agent, indole can play an important role in maize defense priming, preparing systemic tissues and/or neighboring plants for an impending attack [58].

Ancillary data was acquired supporting the premise that bacteria-borne indole may be influencing host metabolism and root physiology. First, root behavior related to auxin (root gravitropism) was investigated due to the genotypic differences in the bacterial mutant strains’ ability to produce auxin. It was noted that FP10, *ipdC* and HM053 strains all exerted similar reductions in root gravitropism (Fig. 5A), a phenotype highly dependent on auxin biosynthesis and/or sensing in the root tip [59]. Because this was elicited by all bacteria strains, it is suspected that infusion of bacteria-borne indole, rather than auxin, may offset auxin homeostasis at the root tip. All functional mutants used in the present study should produce indole, while solely *ipdC* is deficient in the major pathway of auxin formation from indole.

Auxin and ethylene biosynthesis are mutually intertwined [60] with auxin exerting control over 1-aminocyclopropane-1-carboxylate oxidase (ACC oxidase), the controlling enzyme in ethylene biosynthesis. ACC oxidase is a member of the non-heme Fe^2+^ dependent family of oxygenases and oxidases in higher plants [61]. Hence, there could be a strong connection between plant auxin regulation and iron assimilation through the auxin-ethylene relationship. Indeed, it was noted that treatment of plants with exogenous auxin increased uptake and allocation of Fe^3+^ and Fe^2+^ (Fig. 1A, B). In root volatile emissions measurements all functional mutants caused significant reductions in host root ethylene emissions relative to non-inoculated controls (Fig. 5B). This behavior is similar to previously reported results using the endophyte *Herbaspirillum seropedicae* (strain SmR1) in rice [62]. Past published work suggests that PGPB harboring ACC deaminase, the bacteria enzyme that breaks down ACC, lower host ethylene emissions [36, 63], yet physiology studies consistently predict that lower ethylene levels increase plant sensitivity to environmental stress [64, 65]. While there may be some truth to both sides of this paradox, we believe the story is more complex than a simple relationship between microbial ACC deaminase, plant ethylene regulation and plant growth. For instance, it has been previously documented that some members of the PGPB family *Azospirillum* sp. do not possess the ACC deaminase gene [66, 67]. This is true of *A. brasilense*, yet our work demonstrates that root ethylene emissions are significantly reduced with these *A. brasilense* mutants. Furthermore, we contend that the relationship between microbial ACC deaminase and plant growth promotion may not be generalizable across all PGPB interactions since HM053 *A. brasilense* was noted to promote plant growth and, in fact, increase crop yield in maize [10]. Instead, we suggest host ethylene regulation could be coupled to microbial indole synthesis, the precursor for auxin. In the present work, measurements of root indole levels showed that all three functional mutants of *A. brasilense* significantly elevated indole emission rates relative to non-inoculated control plants (Fig. 5C). We suspect this effect could be attributed to the trafficking of bacteria-borne indole into the host root noting that while *ipdC* is deficient in producing auxin, its upstream metabolic machinery for producing indole should be intact. In fact, *ipdC* elicited a higher elevation in root indole emission than HM053 or FP10 inoculants.

There are numerous examples in the literature attesting to the complex interaction between ethylene and auxin [49, 68–70]. Studies have shown that ethylene stimulates auxin biosynthesis and upregulates the transcription of several auxin transporters including, *PIN1, PIN2, AUX1* [71–73]. Ethylene-induced auxin production is localized in the root tip [73] and the auxin signal is subsequently redistributed upward through polar auxin transport. We propose that microbial indole infusion in the upper proximal zones of the roots where the bacteria tend to colonize [4] could locally boost auxin biosynthesis in these tissues thereby impairing auxin biosynthesis in the root tip by disrupting its natural redistribution gradient, and thereby causing a reduction in ethylene emission. This theory is supported by the fact that all three functional mutants caused a significant decrease in root gravitropic response – an action that is linked to root tip auxin regulation.

Finally, HM053 and FP10 inoculants significantly elevated levels of root DIMBOA (Fig. 5D) while *ipdC* did not. The data presented in this figure represents the sum of the endogenous root pool and surface-bound exudate. The influence of HM053 and FP10 on root DIMBOA may, in part be due to the increased indole pool caused by the bacteria, but then the lack of a response from *ipdC* cannot be explained. A theory here is these functional mutants may influence the partitioning of host carbon resources between DIMBOA and auxin biosynthesis, but that remains to be tested in future radiotracer studies. We note that while DIMBOA is typically considered part of maize’s natural defense mechanism, it can also play a role as a Fe^3+^ chelating agent for promoting plant uptake of iron [56]. The observed increase in root DIMBOA levels with HM053 and FP10 inoculation may, in part, contribute to observed elevations in ^59^Fe^3+^ assimilation with these inoculants.

**Fig. 6:**
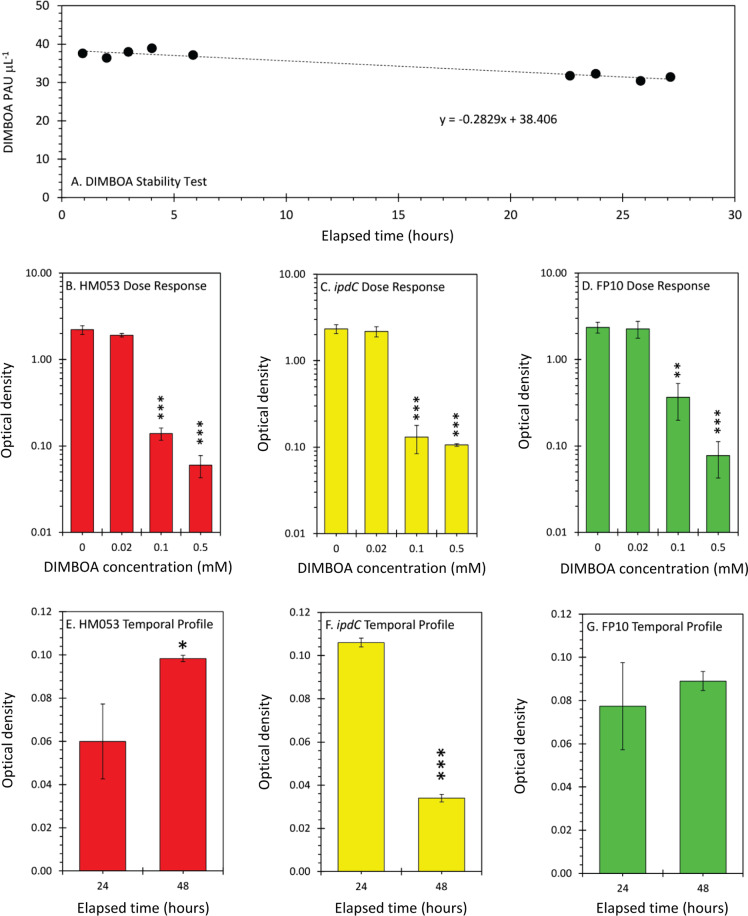
In vitro chemotaxis assays to examine effects of DIMBOA on bacteria growth. **A** DIMBOA stability in the bacteria growth medium was examined using 0.5 mM DIMBOA. Gas chromatography peak area units (PAU) reflecting DIMBOA levels at different time points spanning 27 h shows a ~15% loss of DIMBOA integrity. **B**–**D** DIMBOA dose response effects on bacteria growth at 24 h as measured spectrophotometrically where OD_600_ = 1 corresponds to 10^8^ CFU mL^−1^. **E**–**G** Influence of an acute 0.5 mM DIMBOA dose on bacteria growth after 48 h as measured spectrophotometrically where OD_600_ = 1 corresponds to 10^8^ CFU mL^−1^. Data (±SE) reflected N = 3 biological replicates at each DIMBOA dose. Asterisks indicate significant differences of bacteria growth with different DIMBOA doses relative to 0 mM DIMBOA control (*P < 0.05; **P < 0.01; ***P < 0.001).

One question that remains is why *A. brasilense* would promote production of a bactericide that could potentially be harmful to the microorganism’s health. We note that past work has shown that *Pseudomonas putida*, another PGPB, was tolerant of DIMBOA [74]. To test the theory that certain functional mutants of *A. brasilense* were also tolerant of DIMBOA, we conducted a chemotaxis bioassay examining microbial growth at 0 mM, 0.02 mM, 0.1 mM and 0.5 mM doses of DIMBOA added to the growth media. These doses were selected based on earlier work reported with *P. putida* [74]. In parallel with these assays we conducted a DIMBOA stability test over a 27 hr window (Fig. 6A). Spanning 27 h, DIMBOA underwent only 15% degradation. Results of the chemotaxis assay (Fig. 6B–D) revealed that all three functional mutants tolerated DIMBOA doses of 0.02 mM, but their growth was significantly reduced at higher doses. Using ^11^C data from the present work (Fig. 4B), we used ~30% as an upper limit of root allocation of fixed ^11^C over a 3 h period in the presence of either HM053 or FP10 bacteria. These same bacteria resulted in ~1% fixed ^11^C as acidic exudates over the same 3 h period. Assuming that DIMBOA was the dominant acidic exudate, we estimate that it could account for 0.3% of the total root DIMBOA that was measured in the present work (Fig. 5D) which for HM053 and FP10, could be as high as 10 μg gfw^−1^ or 0.150 μM accumulated over 3 h. Adjusting for 24 h of accumulation and for 15% loss due to degradation, we estimate DIMBOA exudation in the present system could be as high as 1 μM. That said, all three functional mutants tolerated DIMBOA at ~20-times that level. We also conducted a temporal profile of bacteria growth testing the effects of an acute 0.05 mM dose of DIMBOA over 48 h (Fig. 6E–G). HM053 showed a significant increase in bacteria content even at this acute dose over 48 h while *ipdC* showed a significant loss in content, and FP10 remained constant (suggesting that its rate of growth matched the rate of cell death). Hence both HM053 and FP10 functional mutants, which were shown to promote DIMBOA levels within the maize roots, appeared to tolerate the substrate.

Future studies will examine *A. brasilense* growth patterns and demand for plant-borne carbon resources as a function of different DIMBOA doses in the growth media. Recently, we published a method that describes using a combination of C-11 radiographic imaging, gamma counting and optical fluorescence imaging with RAM10 *Herbaspirillum seropedicae*, a green fluorescence reporting strain of an endophytic PGPB, to enable for the first time precise quantification of plant-borne carbon assimilation by a root-associating microorganism [75]. Efforts are underway to generate a stable fluorescent reporting strain of *A*. *brasilense* that will enable us to translate this method to this bacterium and test its tolerance for DIMBOA.

Altogether, the results reported herein show that inoculation of maize seedlings with HM053 *A. brasilense* bacteria boosts host iron assimilation and translocation to aerial portions during early stages of development. This behavior was seen to persist throughout the growing season having beneficial effects on plant growth that resulted in significant increases in crop yield and seed iron content [10]. Thus, the use of beneficial microbes not only influence plant growth, but also can promote crop nutritional content.

Regarding the mechanisms of action, the data presented indicates there are strong influences of bacterial BNF and auxin producing capacities on Fe^3+^ and Fe^2+^ assimilation and *in planta* translocation. While it is enticing to suggest HM053 endows *Strategy I* iron capabilities in maize, the possibility cannot be ruled out that bacteria metabolism may influence the supplied iron oxidation state. However, we note that in the radiographic images taken of aerial portions from ^59^Fe^3+^ and ^59^Fe^2+^ exposures in HM053 inoculated plants (Fig. 2E, F) that there was a different spatial patterning of radioisotope across the leaf surfaces. This observation suggests that the iron oxidation states were not altered by the microorganisms. Future studies, however, will examine this aspect more thoroughly using ^59^Fe^3+^ and ^59^Fe^2+^ coupled with ion chromatography to enable measurement of bacterial uptake and/or manipulation of the different oxidation states of iron as a function of their BNF and auxin biological capabilities.

Supplementary information is available at ISME’s website.

## Supplementary information

Supplemental Information

## Data Availability

All data needed to evaluate the conclusions in the paper are present in the main text or the supplementary materials.
