# Supplementary material for: Functional mutants of Azospirillum brasilense elicit beneficial physiological and metabolic responses in Zea mays contributing to increased host iron assimilation
Source: ISME J. 2021 Jan 6;15(5):1505–22. doi: 10.1038/s41396-020-00866-x (PMC8115672; doi:10.1038/s41396-020-00866-x)
Supplement: Supplementary file 1 — Supplemental Information [file 41396_2020_866_MOESM1_ESM.pdf]

Supplemental Information

Functional mutants of *Azospirillum brasilense* elicit beneficial physiological and metabolic responses in *Zea mays* contributing to increased host iron assimilation

A. Housh, G. Powell, S. Scott, A. Anstaett, A. Gerheart, M. Benoit, S. Waller, A. Powell, J. M. Guthrie, B. Higgins, S. L. Wilder, M. J. Schueller, R. A. Ferrieri

SUPPLEMENTAL METHODS

Production and Administration of Radioactive $^{11}CO_2$: $^{11}CO_2$ ($t_{½}$ 20.4 min) was produced on the GE PETrace Cyclotron located at the Missouri Research Reactor Center using high-pressure research grade N_2 gas target irradiated with a 16.4 MeV proton beam to generate ^{11}C *via* the $^{14}N(p,\alpha)^{11}C$ nuclear transformation (42, 43). The $^{11}CO_2$ was trapped on the molecular sieve, desorbed, and quickly released into an air stream at 200 mL min^{-1} as a discrete pulse for labeling a leaf affixed within a 5 x 10 cm lighted (560 μmol m^{-2} s^{-1}) leaf cell to ensure a steady level of fixation. The load leaf affixed within the cell was pulse-fed $^{11}CO_2$ for 1 min, then chased with normal air for the duration of exposure. A PIN diode radiation detector (Carroll Ramsey Associates, Berkeley, CA 94710, USA) attached to the bottom of the leaf cell enabled continuous measurement of radioactivity levels within the cell during the initial pulse and in the minutes directly following to give information on $^{11}CO_2$ fixation and leaf export of ^{11}C-photosynthates (44).

[11/12C]Metabolite Analyses: [11C]-Sugars were analyzed by radio thin layer chromatography (TLC) using glass backed NH_2-silica HPTLC-plates (200 μm, w/UV254) purchased from (Sorbent Technologies, Atlanta, GA, 30071, USA) according to published procedures (45-47). In the same method, the [12C]-sugars could be measured after thermal reaction of the carbohydrates with amino groups according to prior published work (45). TLC plates were spotted with sugar standards using a semi-automatic Linomat 5 sample applicator (Camag Scientific Inc., Wilmington, NC, 28401, USA) for correlation of biological samples to absolute concentration and developed using a mobile phase consisting of 65:20:15 Acetonitrile: Methanol: Deionized Water (v/v). The TLC plates were imaged using autoradiography and activity in each spot was quantified using ImageQuant TL 7.0 software. Total [11C]sugar content was related to the ^{11}C-activity quantified along the sample lane of the TLC plate, and then corrected to percent total fixed $^{11}CO_2$ using gamma count data from the insoluble and soluble fractions.

[11C]Amino acids were analyzed following our published procedures (45) using pre-column OPA derivatization and quantified by gradient radio HPLC (Sonntek, Inc. Upper Saddle River, NJ 07458, USA) using a Phenomenex X-Bridge 5μm C18 (150 mm x 4.6 mm inner diameter) column heated to 30 °C and mobile phase system comprised of Solvent A (water, pH 5.75), Solvent B (0.01M NaH2PO4, pH 6.8) and solvent C (methanol) starting at 75:25 of A:B at injection and ramping to 20:80 of B:C within 30 minutes at a flow rate of 0.7 mL min^{-1}. On-line fluorescence detection (340 nm excitation, 450 nm emission; Hitachi LaChrom Elite L-2485; Sonntek, Inc.) was used for quantification of the OPA-derivatized [12C]amino acids and a NaI (PMT) gamma radiation detector (Ortec, Inc.) enabling direct measurement of [11C]-labelled

amino acids. Data was acquired using PeakSimple™ chromatography software v4.88 (SRI, Inc., Torrance, CA 90503, USA) and compared to standard calibration curves constructed for each amino acid giving absolute amounts for individual metabolites in units of μmol per gram fresh weight (gfw^{-1}) tissue. Radioactive metabolite peaks were quantified using the same software, corrected for radioactive decay and related back to absolute amounts of fixed ^{11}C activity in the plant at the start of the study.

Metabolite Specific Activity Measurements: Specific activities (SA) of individual metabolites of interest including [^{11}C]histidine, [^{11}C]citric acid and [^{11}C]nicotianamine were calculated using equation 1:

$$SA = (\%\ \text{Fixed}\ ^{11}C\ \text{Activity}) / (\mu\text{moles of metabolite mass} \times gfw^{-1}\ \text{tissue}) \qquad [1]$$

Raw radioactivity counts were decay corrected back in time to the end of the ^{11}C cyclotron target irradiation using equation 2:

$$A_o = A_T \times \exp(\lambda T) \qquad [2]$$

where Ao is the calculated decay corrected radioactivity at T_o or EOB, A_T is the measured radioactivity at time T, λ is the decay constant equal to ($\ln 2/t_{½}$) where $t_{½}$ is the half-life for ^{11}C equal to 20.4 min., and T is the elapsed time from EOB to when the sample was counted. PeakSimple peak integrals for radioactivity amounts were correlated to disintegrations per minute units of absolute radioactivity using a cross calibration factor to relate the HPLC detector response to that of the static NaI (PMT) gamma counter. Metabolite masses in μmoles were calculated from PeakSimple mass peak integrals using the mass detector response to authentic standards.

Autoradiography: Subsequent to Fe-59 exposures plants were harvested, roots were dried and radiographic images of different tissue areas (roots and shoots) were acquired by exposing phosphor plate films for up to 16 hours. Phosphor plates were read using the Typhoon 9000 imager (Typhoon™ FLA 9000, GE Healthcare, Piscataway, NJ 08854, USA). Images were used qualitatively for determining spatial patterning of tracer in roots and shoots.

Transmission Electron Microscopy: Unless otherwise stated, all reagents for electron microscopy were purchased from Electron Microscopy Sciences (EMS, Inc., Hatfield, PA 19440, USA) and all specimen preparation was performed at the Electron Microscopy Core Facility, University of Missouri. Tissues were fixed in 2% paraformaldehyde, 2% glutaraldehyde in 100 mM sodium cacodylate buffer pH=7.35. Fixed tissues were rinsed with 100 mM sodium cacodylate buffer, pH 7.35 containing 130 mM sucrose. Secondary fixation was performed using 1% osmium tetroxide solution containing 1.5% potassium ferrocyanide in cacodylate buffer using a Pelco Biowave (Ted Pella, Inc., Redding, CA 96003, USA) operated at 100 Watts for 1 minute. Specimens were next incubated at 4 °C for 1 hour, then rinsed with cacodylate buffer and distilled water. En bloc staining was performed using 1% aqueous uranyl acetate and incubated at 4 °C overnight, then rinsed with distilled water. A graded dehydration series was performed using ethanol, transitioned into acetone, and dehydrated tissues were then infiltrated with a 1v/1v of Epon and Spurr resin for 24 hours at room temperature and polymerized at 60 °C overnight. Sections were cut to a thickness of 80 nm using an ultramicrotome (Ultracut UCT, Leica Microsystems, Germany) and a diamond knife (Diatome, Hatfield PA 19440, USA). Thin

sections were stained using Reynold's Lead Citrate. Images were acquired with a JEOL JEM 1400 transmission electron microscope (JEOL, Peabody, MA 01960, USA) at 80 kV on a Gatan Ultrascan 1000 CCD (Gatan, Inc., Pleasanton, CA 94588, USA).

Bacteria Quantification - Drop Plate Assay: Bacterial quantifications were performed concurrently with metabolite studies. Plants were harvested from the growth media and rinsed in DI water. Two separate 1-1.5 inch sections of primary root growth were taken from each plant and weighed (approximately 100-300 mg total). The sample was ground with mortar and pestle in 1 mL of 1% saline. Five serial dilutions were performed; the first with 100 μL of the ground extract into 900 μL of 1% saline and each subsequent dilution being 100 μL of the previous dilution into 900 μL of 1% saline. Each serial dilution was plated in triplicate by 10 μL drops onto agar plates fortified with lactate growth media and incubated at 30 ^{o}C for 48-72 hr. before counting. The dilution that contained 3-40 colony forming units (CFU) per 10 μL drop was counted and used to perform calculations (fig. S4).

Root pH Visualization: A solution of Hoagland's nutrient salt (1.608 g L^{-1}) with MES (0.55 g L^{-1}) was prepared in de-ionized H_2O and adjusted to pH of 6.0 with 30% KOH. Gelrite™ was added (2.8 g L^{-1}) and the resulting solution was autoclaved. Once autoclaved, bromothymol blue (0.1196 g L^{-1}) was added. Maize seedlings were germinated as described and transplanted to glass cells containing the dye enriched gel mixture. After five days of growth seedlings were removed and imaged with a digital camera (fig. S5).

Principal Component Analysis of ^{59}Fe Data: The information included in our $^{59}Fe^{2+/3+}$ allocation measurements were represented by two principal components (PC), with PC1

representing 86.07% of the information embedded in the data and PC2 13.93%. The PCs selected to represent the data are classified as feature vectors (F1 and F2) as shown on the biplot. The axes are in terms of the eigenvalues, with larger values indicating a greater variance, thus a greater representation of the information within the data. The active variables shown in gray represent the initial variables of allocation of Fe^{2+} and Fe^{3+} in maize. The length of the active variable vectors indicates how well the variables are tied to the feature vectors. Since both the active variable vectors are equivalent in length and are found equally between F1 and F2 it can be interpreted that both active variables are equally represented by both F1 and F2.

SUPPLEMENTAL FIGURE LEGEND

Supplemental fig. S1. Mechanisms for higher plant assimilation of iron from soil. Non-gramineaceous plants typically follow a Strategy I mechanism in which a proton-ATPase enzyme on the root epidermis secretes protons into the rhizosphere to acidify the soil and solubilize the ferric salts which are typically present as silicates and oxides. Ferric reductase oxidases (R) reduce the ferric to ferrous ions where protein transporters (TR) facilitate uptake into the root cells. Graminaceous plants rely on a Strategy II mechanism whereby they biosynthesize specialized phytosiderophores (PS, or chelating agents) and excrete (E) them into the soil to complex with the ferric iron enabling the roots to take up this chelated form with the aid of specialized transporters (Tr).

Supplemental fig. S2. Maize plants raised for ^{59}Fe studies were grown aeroponically in a commercial growth chamber. Growth conditions were set for a 12 hr. photoperiod at 500 µmol m^{-2} s^{-1} lighting and a day/night temperature of 25/20 °C; relative humidity of 70-80%. Hoagland's basal salt mix nutrient (pH 6.0) was introduced when plants were transplanted to the system.

Supplemental fig. S3. Maize plants raised in Turface for ^{11}C radiotracer studies. Growth conditions were set for a 12 hr. photoperiod at 500 µmol m^{-2} s^{-1} lighting and a day/night temperature of 25/20 °C; relative humidity of 70-80%. 50 mL of Hoagland's basal salt mix nutrient (pH 6.0) was introduced every 5-days.

Supplemental fig. S4. Drop plate assay analysis on the extent of bacteria colonization of maize roots. Roots were inoculated for 1 hr with *ipdC*, FP10 and HM053 strains of *A. brasilense* at the 5 day old seedling stage and then transplanted to their respective growth media (aeroponics or solid Turface™). Two separate cohorts of plants were tested with the different strains of bacteria. Data (±SE) reflects N=4-6 biological replicates. Asterisks indicate significant differences of treatment compared to controls (*P<0.05).

Supplemental fig. S5. Visual assay of root pH in 3 day old maize seedlings reveals a higher level of acidic pH surrounding HM053 and FP10 inoculated roots. Visualization was acquired by adding bromothymol blue pH indicating dye to Gelrite™ semi-solid growth media. Areas

on the root showing a red coloration are indicative of acidic pH. Seedlings were extracted at 3 days, washed and digital photos taken.

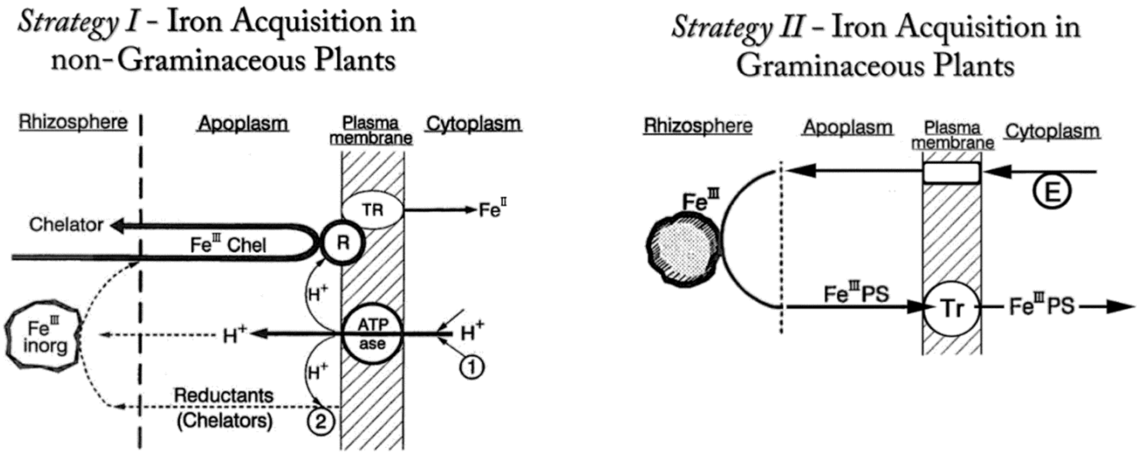

Supplemental fig. S1.

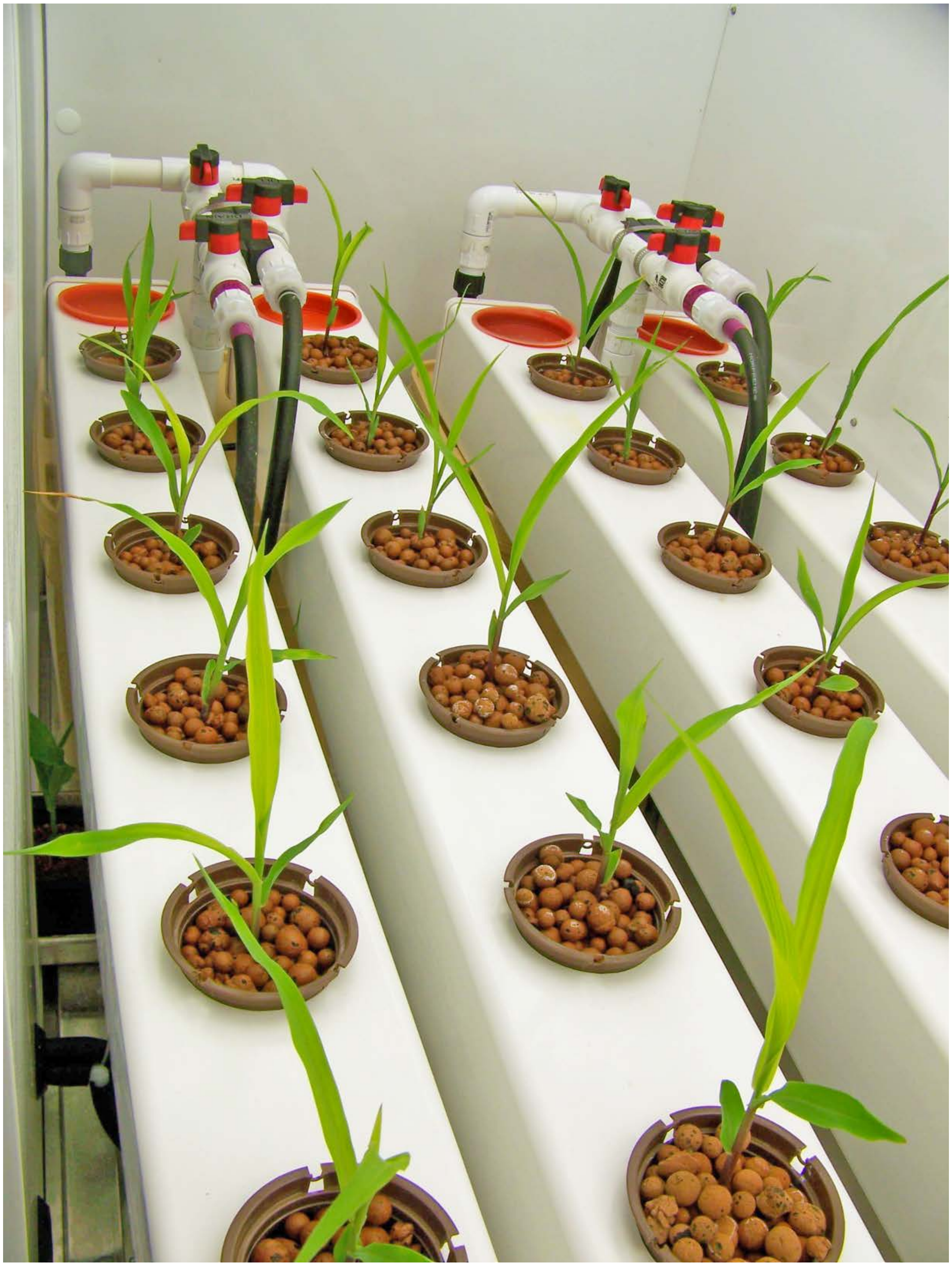

Supplemental fig. S2.

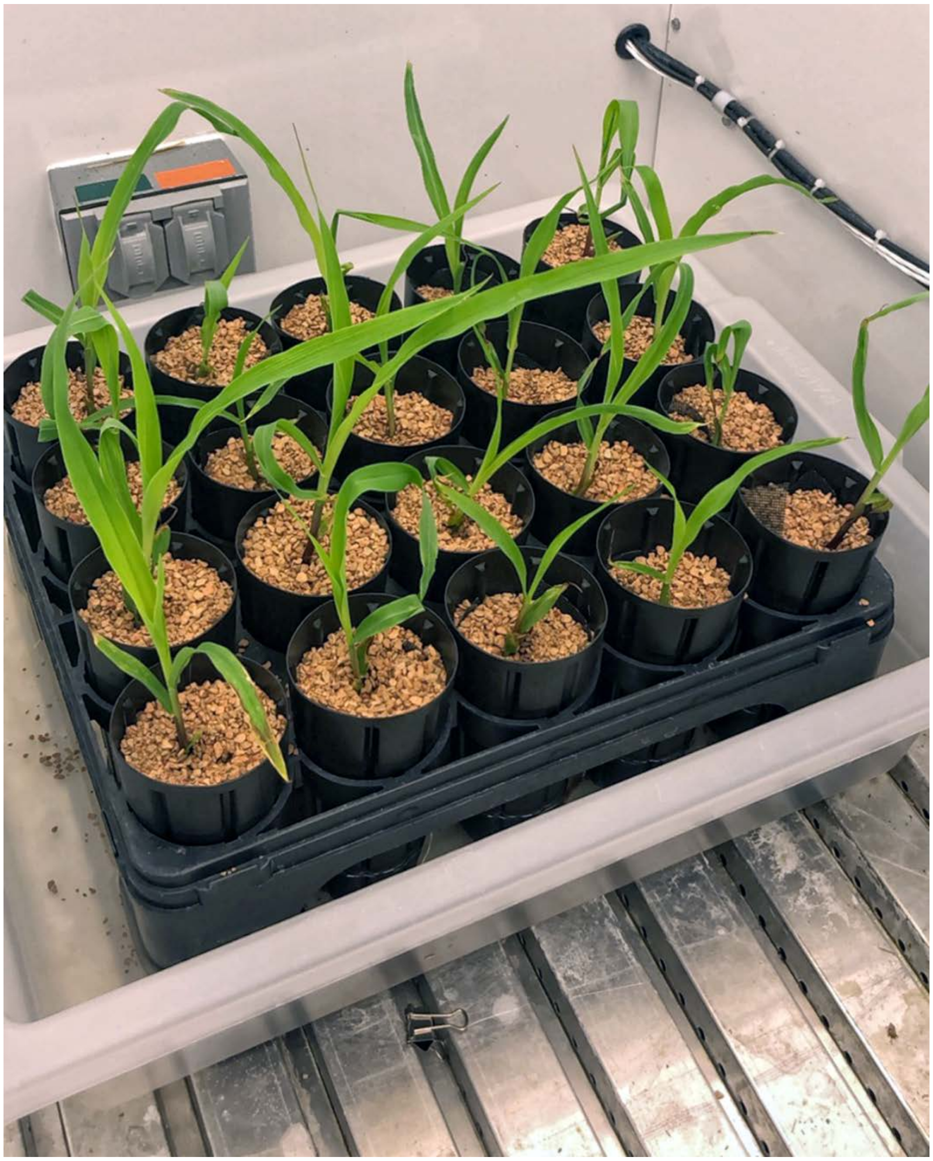

Supplemental fig. S3.

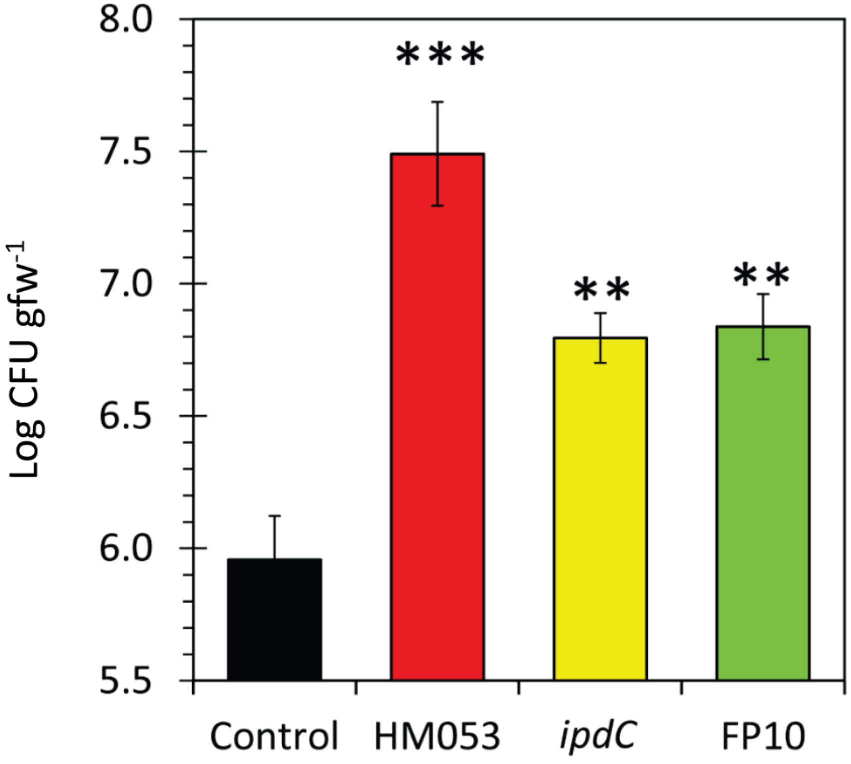

Supplemental fig. S4.

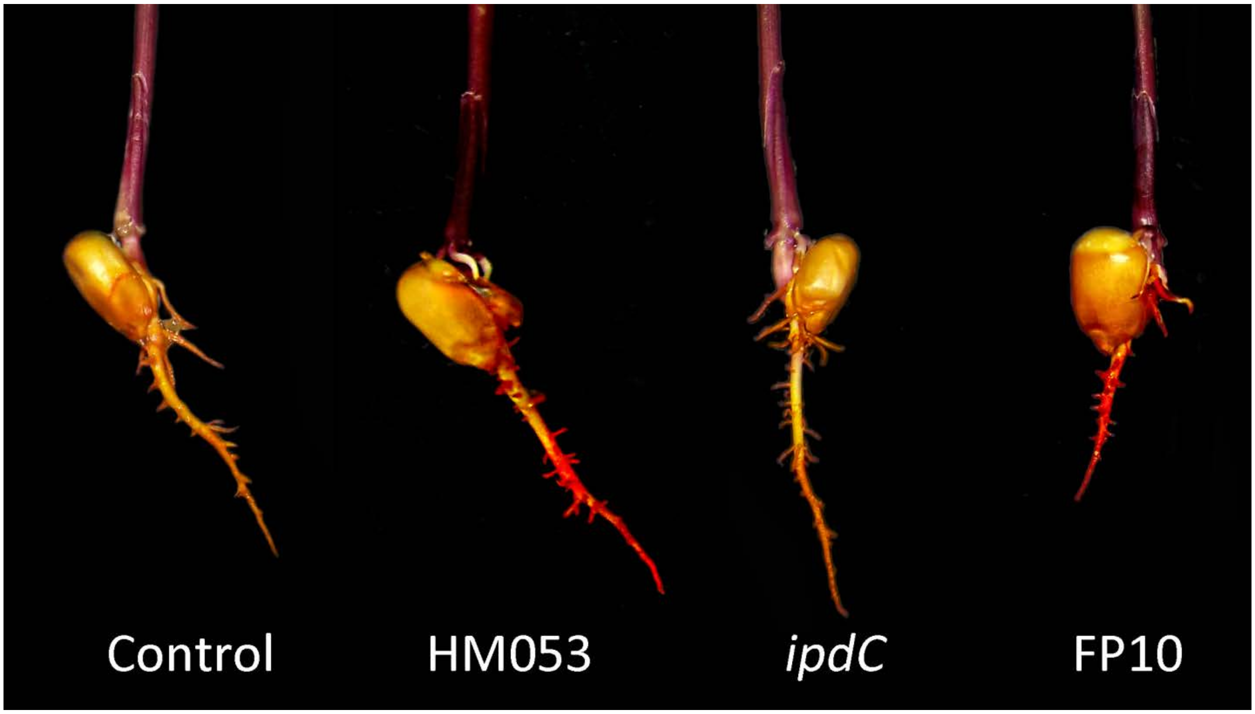

Supplemental fig. S5.
